# Reconstruction of reaching movement trajectories using electrocorticographic signals in humans

**DOI:** 10.1371/journal.pone.0182542

**Published:** 2017-09-20

**Authors:** Omid Talakoub, Cesar Marquez-Chin, Milos R. Popovic, Jessie Navarro, Erich T. Fonoff, Clement Hamani, Willy Wong

**Affiliations:** 1 Department of Electrical and Computer Engineering, University of Toronto, Toronto, Canada; 2 Institute of Biomaterials and Biomedical Engineering, University of Toronto, Toronto, Canada; 3 Rehabilitation Engineering Laboratory, Toronto Rehabilitation Institute–University Health Network, Toronto, Canada; 4 Neural Engineering Laboratory, Toronto Rehabilitation Institute–University Health Network, Toronto, Canada; 5 Division of Functional Neurosurgery of Institute of Psychiatry, Hospital das Clínicas, Department of Neurology, University of Sao Paulo Medical School, São Paulo, Brazil; 6 Toronto Western Research Institute–University Health Network, Toronto, Canada; Shanghai Jiao Tong University, CHINA

## Abstract

In this study, we used electrocorticographic (ECoG) signals to extract the onset of arm movement as well as the velocity of the hand as a function of time. ECoG recordings were obtained from three individuals while they performed reaching tasks in the left, right and forward directions. The ECoG electrodes were placed over the motor cortex contralateral to the moving arm. Movement onset was detected from gamma activity with near perfect accuracy (> 98%), and a multiple linear regression model was used to predict the trajectory of the reaching task in three-dimensional space with an accuracy exceeding 85%. An adaptive selection of frequency bands was used for movement classification and prediction. This demonstrates the efficacy of developing a real-time brain-machine interface for arm movements with as few as eight ECoG electrodes.

## Introduction

A brain-machine interface (or BMI) uses brain signals as a means of controlling external devices. In recent years, the scientific community has focused considerable efforts in developing BMI systems that would allow a person with an amputation or with a high-level spinal cord injury to control complex prosthetic devices or multi-degree-of-freedom robotic arms [1–5].To generate control commands for such a device, it is necessary to detect several control parameters. These parameters can include: (i) movement onset; (ii) direction of movement; (iii) desired end-position of the arm and/or hand; (iv) position over time; (v) velocity; and (vi) grasping posture to be performed once hand reaches the object. This paper is concerned with the rapid and accurate determination of movement onset and movement trajectory.

Ideally, these parameters are detected before movement initiation with sufficient lead-time to execute the desired reach/grasp movement. One approach to detecting ‘discrete’ states like onset, direction or grasp posture is to identify templates of brain activity that can then be used for classification, e.g. [6]. However, shortcomings of this approach include having to fix a priori the number of gestures used, as well as not being able to specify the parameterization of the movement, e.g. the choice of speed or force [6–8]. Most of the studies employing template matching are based on electroencephalographic signals (EEG).

Continuous parameters like hand position or velocity over time can be better addressed by defining a functional relationship which maps brain activity to movement kinematics. Such mappings can then be used to drive a prosthetic or robotic arm. A number of studies have demonstrated successful control of robotic arms with up to 10 degrees of freedom [7–21]. Typically, these systems use single or ensemble neuron activities acquired via intra-cortically implanted microelectrodes, and have evolved largely from the original work of Georgopoulos et al. where they showed that movement direction can be reliably detected from single-unit activity [9]. There are, however, important limitations associated with single unit BMI’s, including the highly invasive nature of these recordings, as well as the need for constant system calibration and tuning.

To address these issues, one way is to look at other recording paradigms. Electrocorticography (ECoG) is a minimally invasive method for measuring brain activity compared to intracortical methods because the electrodes do not penetrate brain tissue. ECoG uses macroelectrodes placed just over (or under) the dura matter and the signals are considered to have larger bandwidth and higher spatial resolution than EEG [13,19]. In particular, gamma activity (>30 Hz) can be readily observed in ECoG recordings. This paper focuses on an ECoG-based approach with the goal towards decoding movement onset and execution.

A number of studies have explored the neural correlates of movement planning. In an EEG study, Kornhuber and Deecke [22] found the presence of a slow, negative potential as early as 1.5 seconds before execution of self-initiated movement. More recently, movement onsets were found to be detectable from EEG using slow components 500 ms prior to movement onset [23–25]. Slower oscillations in EEG recordings tend to be more informative than the higher frequency components because faster oscillations suffer from low signal-to-noise ratio and low spatial resolution. By contrast, ECoG recordings do not have these problems and one can reliably observe the onset of beta and gamma activity over the motor cortex prior to the commencement of movement [26,27]. Despite this, there have been far fewer attempts at detecting movement onset from ECoG signals. For example, Grainmann et al. used changes in beta or delta activity [8]. While their results were encouraging their method relies on template matching delaying the detection process by about 1 second. More recently, Wang et al. used a support vector machine classifier to detect the movement periods from spectral density of the ECoG activity [27]. Classification was based on temporal and spatial patterns of activity observed across multiple (50+) ECoG electrodes with extensive coverage of neocortical regions making such a system not practical for clinical use.

As such, there remains many challenges in developing a truly functional ECoG-based BMI system. The majority of studies discussed above use ECoG electrodes with a large number of contacts, with researchers “handpicking” a subset of electrodes providing the most informative inputs [13,14,28–30]. Moreover, few studies have been able to demonstrate decoding of complete arm kinematics (both movement onset and velocity) from ECoG activity. Instead, the focus is generally on singular aspects of movement detection like onset or direction only. Several earlier studies have investigated continuous, short-ranged movements like joystick manipulation using regression models of ECoG activity [12–14]. But it remains unclear if ECoG signals can be used to predict upper limb kinematics with accuracies comparable to the ones achieved using microelectrode recordings.

The main objective of this study is therefore to develop an end-to-end ECoG-based BMI system that can predict movement onset and trajectory for gross arm movements using a small number of clinically realizable ECoG contacts implanted over the primary sensorimotor cortex.

## Materials and methods

### Participants

Three participants from the Functional Neurosurgery Clinic at the Hospital das Clínicas of the University of São Paulo, Brazil, were recruited for this study. Participants 1 and 3 were men of 51 and 42 years of age respectively. Participant 2 was a 48 year-old woman. They were implanted with ECoG electrodes over their primary cortex as part of the treatment for chronic pain using direct cortical stimulation.

### Electrocorticographic (ECoG) electrode implantation

**Fig 1:**
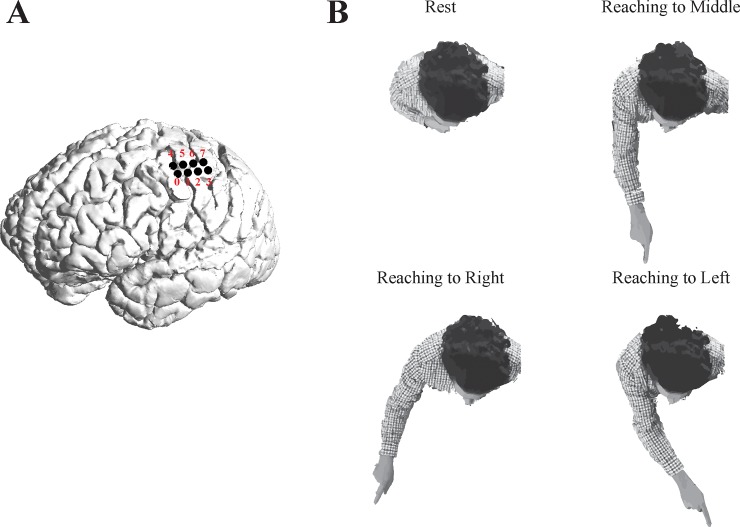
A) **Location of implanted ECoG contacts shown for Participant 2.** Location of ECoG electrodes was identical for all participants. Contacts for the first strip were labeled 0–3 from distal to proximal relative to the electrode connector, and contacts of the second strip were similarly indexed 4–7. The functional location of Contact 1 was confirmed by electrically stimulating the cortex and observing finger or wrist movements of the contralateral upper limb. B) Movements performed by the participants of the study: reaching a target placed 30 cm to the middle, right, and left of the individual's midline.

All participants were implanted with epidural electrodes over their sensorimotor cortex as part of a pain management system. The procedure was identical for all participants. A small craniotomy was done under local anesthesia and sedation. Once the bone flap was removed, specific motor regions were identified either by using transdural electrical stimulation or through the recording of motor evoked potentials [31]. Thereafter, two paddle leads were implanted in the epidural space (Lamitrode 3240 St. Jude Medical Inc., U.S.A.), perpendicular to the motor strip over the regions where contractions were evoked or motor evoked potential recorded (see Fig 1). Each paddle consisted of a single row of four platinum discs embedded in a silicon membrane. The electrode contact was 4 mm in diameter and the center-to-center distance between adjacent contacts was 10 mm.

The four electrode contacts of the first strip were numbered 0–3 (distal to proximal) relative to the electrode connector. Contacts 0–1 (the first and second contacts of the first strip) were located over the primary motor cortex. The functional location of Contact 1 was confirmed by electrically stimulating the cortex (monopolar pulses with frequency 50 Hz, pulse width 100 μs, and amplitude 3–10 μA) and observing the resulting finger or wrist movements on the contralateral upper limb. Contacts 2–3 were situated with partial coverage over the primary sensory cortex. The second strip was similarly labelled 4–7 (distal to proximal) relative to the connector. This strip was placed parallel to the first strip such that Contact 5 (the second contact of the second strip) was positioned over the primary motor cortex. Contact 4 was over the motor cortex and 6 over the primary sensory cortex. Contact location was identical for all subjects. Fig 1 shows the location of the implanted electrodes for Participant 2 by cross-referencing a MRI image taken prior to electrode implantation with a CT image taken after the surgery.

The participants had the electrode leads externalized for 6 days following the implantation to allow a neurologist to select optimal stimulation parameters (polarity, amplitude, frequency, duration) before the entire system, including the stimulator, was internalized permanently. We conducted our study during the time period in which the leads were still externalized. The study was approved by the University of Sao Paulo Research Ethics Board, and all participants provided signed informed consent prior to taking part in the experiments.

### ECoG, EEG, and EMG recording methodologies

In addition to ECoG measurements, electroencephalography (EEG) signals were recorded at the C3, C4, Cz, Fz, and Fp1 locations of the 10–20 electrode placement system. The purpose of recording EEG activity was to identify and reject trials contaminated with eye or facial/head movement artifacts. In addition, electromyography (EMG) signals were obtained from the wrist flexors, wrist extensors, biceps, and triceps muscles. The electromyography signals were bandpass filtered between 20–500 Hz. These signals were used to determine movement onset time.

All signals (EEG, ECoG and EMG signals) were recorded using a 16-channel biosignal acquisition device (g.USBamp, g.tec, Austria) with sampling frequency of 1200 Hz. The signals were referenced to the ear lobes and grounded to the clavicle. The recording device had a built-in 8^th^ order digital Butterworth anti-aliasing filter with pass-band frequency range of 0.1 to 500 Hz. To detect movement onset, the activity of the motor cortex was recorded from two ECoG contacts in a bipolar arrangement. For this, we chose Contact 1 as the signal source of relevance for our experiments because it was situated directly above the wrist region (verified using electrical stimulation). Contact 0 was chosen as the reference electrode since it was adjacent to Contact 1, and also located above the primary motor cortex (Fig 1).

### Kinematics recordings

**Fig 2:**
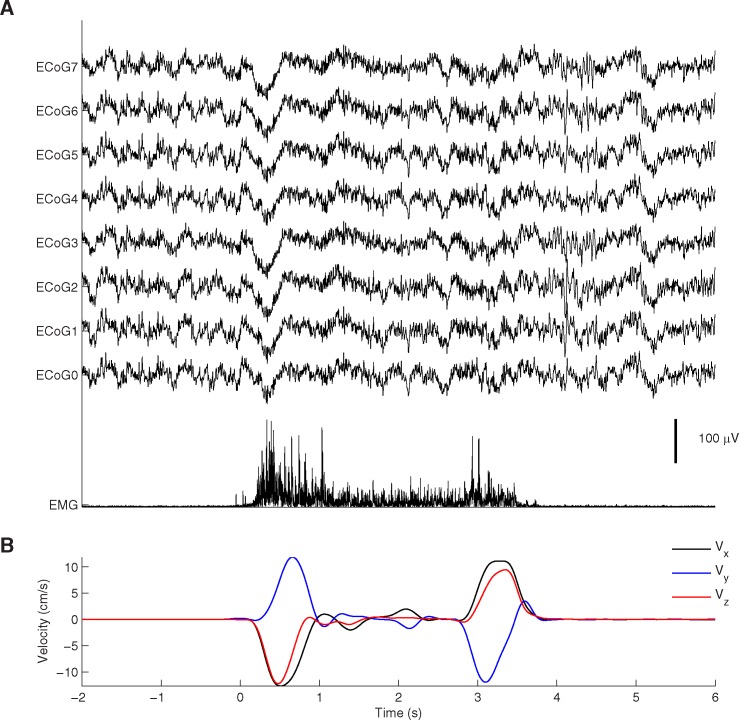
Example of recordings. A) Traces showing raw ECoG signals and EMG recorded during a reaching task for Participant 3. B) Arm velocity in three-dimensions during the task.

The upper limb movements were recorded using a three-dimensional electromagnetic motion capture system (Fastrack, Polhemus Inc, U.S.A.) and a custom-made data acquisition software written in C. The sensor was placed on the index finger. The three-dimensional position of the sensor was recorded with a sampling frequency of 40 Hz and was time stamped. The upper limb kinematics were recorded and synchronized using the same computer that captured the ECoG, EEG and EMG data. An example of recorded signals is shown in Fig 2.

### Experimental trial

We defined an experimental trial as the period beginning 4 seconds prior to and ending 8 seconds after the onset of the reaching task. The trials were aligned with respect to the movement onset, identified by the onset of muscle activity when it exceeded 3 standard deviations of the baseline value. Trials contaminated with eye blinks were identified through visual inspection of the activity in the Fp1 and Fz electrodes and excluded from further analysis.

### Experimental protocol

The participants performed reaching tasks with the arm contralateral to the site of electrode implantation while sitting in a chair. The experimental task involved the participants reaching to targets on the *left* (RTL), *middle* (RTM) and *right* (RTR) placed at the level of their chest between 30–40 cm with respect to the sagittal plane. All targets lie within comfortable reach for all participants. They were asked to fixate their gaze on the middle target during movements.

At the beginning of the task the participants had their hand resting on a pillow placed on their lap (i.e. resting position) which was verified that the arm/hand were relaxed by the absence of EMG activity. The participants received an auditory cue (‘GO‘ signal) to start reaching towards the pre-specified target. After reaching the target successfully, the participants were instructed to wait a few seconds before returning their hand to the initial/resting place. Following this, there was a rest period of randomized duration (6–8 s) before the participants receive a new ‘GO‘ signal to perform the next task. This sequence was repeated until the end of the session when each task was repeated at least 40 times.

### Analysis

We present our analysis in two main sections. First, we identify changes in the spectral density of the ECoG recordings attributed to the movement and use them to identify the movement onset. Second, we show that the activity of the motor cortex can be used to reconstruct the trajectory of the moving arm.

#### Identifying movement onset

A generalized decrease in band power during voluntary movement is known as an *event-related desynchronization* (ERD) and, conversely, an increase in band power is known as an *event-related synchronization* (ERS) [32]. These two responses are measured with respect to a chosen baseline when no movement-related activities are expected (i.e. at rest). ERD and ERS can be observed in specific frequency bands including delta (1–4 Hz), alpha (8–12 Hz), beta (13–30 Hz) and gamma (> 30 Hz). For example, the beta activity is suppressed during movement planning and execution [17,26,32–34], and this decrease of power has been used successfully to detect the onset of movement [6,8]. While it is not uncommon for the entire alpha, beta, or gamma bands to show changes during voluntary movement, usually a narrower set of spectral components specific to each individual shows the greatest response.

Trials were extracted from ECoG recordings for offline analysis and were analyzed in the time-frequency domain. A spectrogram, consisting of a windowed short-time Fourier transform of the signal, was used to determine the frequency content changes in power as a function of time. The ECoG signals were windowed in segments of 0.5 s using a Hamming window. A Fourier transform was then computed for the segment resulting in a spectrum with a resolution of 1 Hz. The window was then shifted forward by 10 ms, and the procedure was repeated until the end of the trial was reached. The resulting spectrogram consisted of a matrix where each column represents the power spectrum of a windowed signal, and each row of this matrix represents the time series of power of the signal at a particular frequency. Event-related changes (ERD/ERS) were calculated by normalizing each row using the power of the baseline signal for that frequency. Baseline is defined as 500ms window of ECoG activity prior to the initiation of movement (between -2 and -1.5 seconds). Baseline values were compared statistically (Kolmogorov-Smirnov test) with the values recorded after the ‘GO‘ cue and during the movement to identify significant differences in power.

Once the spectrograms were generated, we selected frequency sub-bands specific to each participant to detect the onset of movement. We did this by first inspecting the entire frequency content of the ECoG signals in 1 Hz bands and identifying the spectral components with maximum ERD/ERS. The amplitude of these components falls sharply around their peaks (see results for more details). Therefore, we focused on frequency bands specific to each individual to maximize detection of movement onsets. Four-hertz bands, centered at the frequency with maximum ERD/ERS activity, were monitored to assess their suitability for detecting movement onsets (see Table 1). For each trial, we calculated the power in the 200 ms windows with 50 ms overlap. The windows were labelled as “movement” and “no-movement” through visual inspection of muscle activity. We then used the first 10 trials of the experiment to train a linear classifier to detect the onset of movement. The classifier associated the amplitude of bandpass activities with the movement state. We then applied the classifier to the entire (continuous) ECoG recordings. The detection accuracy of the classifier was evaluated using the so-called F1-score [35], which is a normalized measure quantifying classification accuracy in cases where one outcome (i.e. idling) is more probable than a second outcome (i.e. movement). The F1-score has a value between 0 and 1, where 1 denotes perfect classification, and is defined as

$$F1=\frac{2TP}{2TP+FP+FN}$$ (1)

where TP, FP, and FN are the true positive, false positive, and false negatives rates respectively.

**Table 1:** Frequencies where maximum ERD/ERS were observed in the gamma and beta bands. Peaks identified by frequencies at which maximum percentile changes were observed relative to pre-movement baseline level. Bandwidth defined as the width of frequency band where ERS/ERD was above 50% of its peak value.

Participant	α-β peak (Hz)	max α-β -ERD (%)	α-β bandwidth (Hz)	γ peak (Hz)	max γ-ERS (%)	γ bandwidth (Hz)
1	22	87	10	155	281	19
2	24	54	20	64	247	8
3	22	52	22	77	114	14

#### Reconstructing velocity with multiple linear regression

We used multiple linear regression (MLR) to model the relationship between neural and kinematic data. To do this, we assumed that the arm velocity can be reconstructed from the amplitude of activities of low frequency, beta, and gamma bands. MLR models have been used previously to estimate kinematics from brain activities recorded using EEG [36], MEG [37–39], and ECoG [40,41]. The input to our MLR model is a vector containing the sampled time points of the cortical activity measured at each contact which we denote as **E**(t) and the output is 3D arm velocity, **K**(t). More specifically, **E**(t) is a vector containing the raw unfiltered ECoG activity (8 channels), together with the amplitude of the ECoG activity filtered separately for both the alpha-beta (8 channels) and the gamma band (8 channels). The amplitudes for alpha-beta and gamma activity were extracted using an envelope detector together with a low-pass filter set with a cutoff of 5 Hz. In total, **E**(t) is a vector of length 24. The relationship between cortical activity and velocity is given by the equation:

K(t)=b+E(t)β+η(t) (2)

where both **b** and the matrix of regression coefficients **β** are constants estimated by the method of least-squares from our experimental data. The final term in the equation η(t) represents the residual errors.

Although Eq (2) describes the process by which hand velocity can be reconstructed from ECoG activity, it does not take into consideration two important physiological characteristics of the motor system. The first is the delay between the cortical activity (motor command) and the corresponding motor output (movement). This lag represents the transmission delay between the cortical motoneuron activation and observable EMG/kinematic activities, and consists of conduction time in the corticospinal tract, relay in the spinal cord, conduction time of motor axons and the neuromuscular junction [42–45]. We incorporated the delay into the linear model as a fitting parameter.

The second modification required for Eq (2) relates to the ERD/ERS activity in the beta and gamma frequency bands. These events are reliable indicators of the starting and stopping of arm movements [8,27]. Inclusion of these parameters makes it possible to “gate” (i.e. turn on or off) the predicted output **K**(*t*). Specifically, since gamma band activity appears to be well correlated to the absence or presence of movement (see results, [46,47]), increased gamma energy can be used as an indication of movement period. Gating by gamma activity was incorporated through a threshold function, **h(t)**, with Eq (2) rewritten as

$$K(t)=b+\sum_{u=t_{0}}^{t_{1}}h(t-u)E(t-u)\beta (u)+\eta (t)$$ (3)

where *u* is a positive time lag between the cortical activity and the motor output. The thresholding function **h**(t) incorporates the movement onset classifier with gamma activity (detailed earlier) implemented as a Heaviside function. Accuracy of estimated arm velocity was evaluated for each participant through a leave-one-out, a cross-validation method: arm velocity of a single trial was estimated using the remaining trials as a training set. We calculated the Pearson correlation between the estimated and actual limb velocity for each permutation.

## Results

### Detection of movement onset

**Fig 3:**
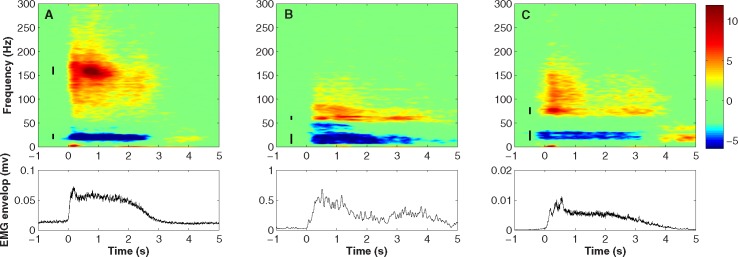
Changes in spectral density of ECoG contact located over primary motor cortex derived from activity of Contact 1. Changes shown in dB with accompanying EMG responses. (A) Response of Participant 1 shows ERS in gamma band (center frequency 158 Hz) and slow oscillations (0–2 Hz) as well as ERD in alpha-beta band (10–30 Hz). Time required for participants to reach target and to return to initial position was approximately 2 seconds. Vertical black lines indicate frequency bands where ERS/ERD was reduced by 50% of its peak value (3 dB drop). (B) Results for Participant 2 and (C) Participant 3.

The power in both the low-frequency (< 2 Hz) and gamma (>30 Hz) frequency bands were elevated during the movement phase. Correspondingly the alpha-beta frequency band (8–30 Hz) was found to decrease (Fig 3). Changes in all three bands were statistically significant for all participants (p < 0.01, Kolmogorov-Smirnov test). The ERD/ERS results are shown in Table 1 where it is evident that the centre frequency and the magnitude of power changes are different for each participant. In all cases however, we observed that the changes in power in the gamma frequency range were higher (> +100%) than either those registered in the alpha-beta or low-frequency bands. Moreover, the frequency bands over which maximal ERD/ERS was found were different for each participant. This is the reason why we focused on a narrowband of frequencies in the gamma band which showed maximum ERD/ERS activity for detection of movement onset.

**Fig 4:**
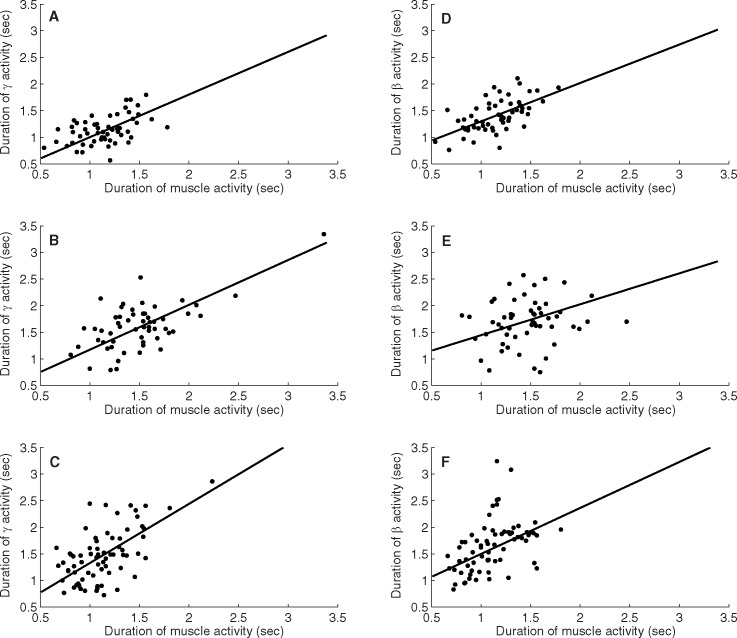
Duration of activity plotted versus EMG activity for Participants 1–3 together with regression lines for gamma band activity (A-C) and beta band activity (D-F).

The results also show a clear relationship between duration of ERS/ERD and movement duration. In Fig 4A–4C, the duration of gamma activity was plotted against duration of EMG activity. A high degree of linear correlation between the two variables can be observed as shown by the regression line. Repeating the analysis with the intercept fixed at zero yields lines with near unity slope and only a slightly decreased goodness of fit (R-squared) (Table 2). This suggests two important observations: (a) the recorded ECoG signals are not simply evoked responses, and are likely to be correlated to control and signaling within the motor system; and (b) gamma activity provides a convenient marker to monitor the commencement and cessation of movement activity.

**Table 2:** Parameters for regression lines relating duration of EMG with length of ERD/ERS during movement. Regression lines also calculated with intercept constrained to zero.

Participant	Frequency Band	Slope (with 95% confidence bounds)	Intercept (with 95% confidence bounds)	R-squared
1	γ (ERS)	0.76 (0.66, 0.86)	0.22 (0.08, 0.36)	0.56
2	γ (ERS)	0.85 (0.73, 0.97)	0.23 (0.064, 0.40)	0.77
3	γ (ERS)	0.95 (0.80, 1.10)	0.048 (-0.08, 0.18)	0.61
1	γ (ERS)	0.97 (0.95, 1.00)	0	0.43
2	γ (ERS)	1.00 (0.96, 1.05)	0	0.74
3	γ (ERS)	1.01 (0.97, 1.05)	0	0.62
1	β (ERD)	0.72 (0.45, 0.99)	0.58 (0.26, 0.89)	0.33
2	β (ERD)	0.58 (0.28, 0.88)	0.86 (0.39, 1.33)	0.22
3	β (ERD)	0.86 (0.56, 1.17)	0.63 (0.28, 0.99)	0.56
1	β (ERD)	1.2 (1.13, 1.27)	0	0.13
2	β (ERD)	1.12 (1.03, 1.21)	0	0.02
3	β (ERD)	1.44 (1.35, 1.53)	0	0.44

Similar fits using alpha-beta ERD and low-frequency ERS show similar trends with overall poorer fit. In particular, the R-squared values using ERD in the beta frequency range were found to be consistently lower for all participants. Results for the low frequency (<2 Hz) component departed from linearity suggesting either issues with reliability or a more complex underlying relationship.

**Fig 5:**
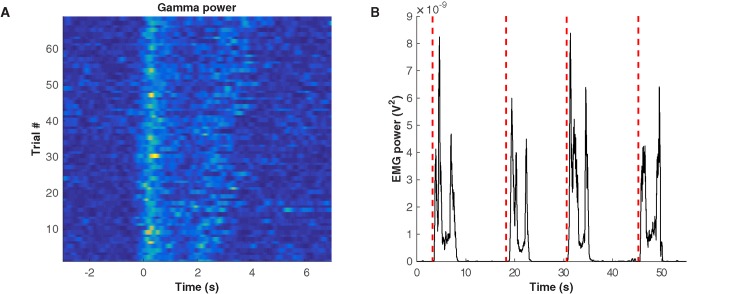
Data of Participant 3. (A) Gamma power (70–90 Hz) during 50 consecutive trials. EMG onsets are aligned at t = 0. Each trial contains both reaching and retrieval movements. (B) Time course of muscle activity and detected movement onsets. Four typical movement cycles are shown. The solid line shows power as calculated from biceps muscle activity (i.e. the square of the EMG signal) and red vertical dashed lines represent detected movement onsets.

The high degree of correlation between movement duration and gamma band activity suggests that ECoG activity can also be useful in detecting movement onset and termination. To do this, we have used linear discriminant analysis using activity from low-frequency (delta), beta and gamma bands. After training and testing with the classifier, results show that gamma activity is the best indicator of movement initiation confirming the results we obtained through regression analysis. Fig 5A displays gamma power during 50 consecutive trials performed by Participant 3. As expected, gamma power is elevated consistently during each reach task.

Moreover, movement onsets were detected with a near-perfect F1-score. Table 3 shows F1-scores obtained for all participants and Fig 5B displays one example of the EMG activity recorded during the experimental trials for Participant 3 along with the predicted onset times. The figure shows repeated trials of the hand reaching towards the target followed by return to the rest position. Predicted movement onsets are shown by the dashed red lines.

**Table 3:** Movement detection through linear discriminant analysis and average correlation coefficient between actual and reconstructed kinematic path for reaching to right (RTR), middle (RTM), and (RTL), as well as average correlation.

Participant	Detection rate	Detection latency	Reconstruction Accuracy			
	F1-score		RTL	RTM	RTR	Average
1	0.99	20±5 ms	0.93±0.06	0.95±0.05	0.97±0.04	0.95
2	0.99	30±8 ms	0.82±0.14	0.91±0.1	0.93±0.09	0.88
3	0.98	25±6 ms	0.73±0.19	0.84±0.12	0.82±0.14	0.80

### Choice of frequency bandwidths

**Fig 6:**
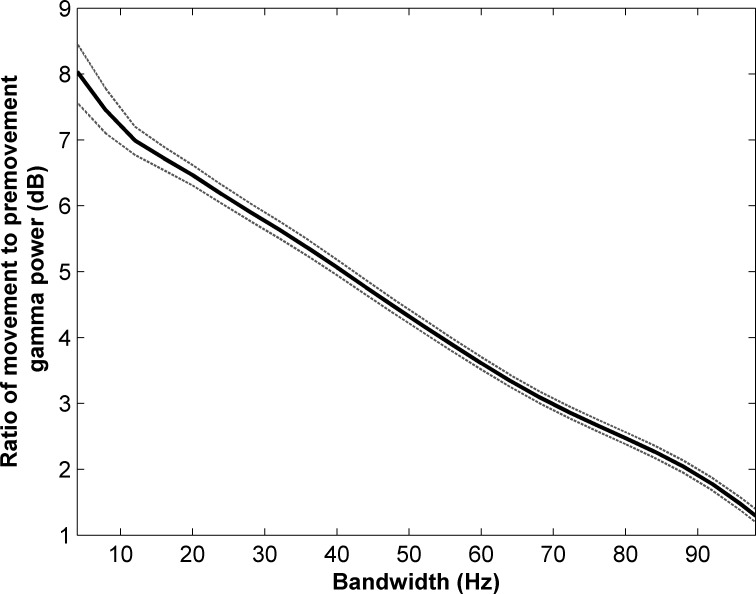
Effect of bandwidth used to calculate the band power for Participant 3. Ratio between movement (0-1sec) and pre-movement (-1 to 0sec) gamma power plotted as a function of bandwidth. Both mean and MSE are shown (solid and dotted lines respectively). The ratio declines monotonically and approaches 1 indicating poor discrimination between movement and rest when using large bandwidths.

The frequencies for maximum changes in movement-related activity (ERS/ERD) were different for each participant. Moreover, we observed that peak activity lie in a comparatively narrow range, with activity falling sharply around its peak (Fig 3 and Table 2). As such, we tailored the frequency band to be specific to each individual. This helped increase sensitivity to detection of movement onsets. To illustrate the effect of bandwidth on detection, we plotted discrimination performance as a function of bandwidth. Bandwidth was increased in 2 Hz increments and the band power calculated in the time domain after filtering with a 3rd order Butterworth filter. The ratio between movement (0–1 sec) and pre-movement (-1 to 0 sec) activity was calculated. A large ratio indicates better discriminability, whereas values close to 1 represent little to no discriminability. Fig 6 shows the ratio plotted in dB as a function of bandwidth. The ratio between the movement and pre-movement gamma power decreases monotonically and approaches 1 with increasing values of bandwidth.

### Reconstruction of velocity from cortical response

Eq (3) postulates that movement velocity can be derived from the amplitude of ECoG activity. The equation parameters are the delay between kinematics and ECoG (*t*_0_), model order (*t*_1_-*t*_0_), and the coefficients (**β**). We used a model with degree of 6, i.e. *t*_1_-*t*_0_ = 5ms, to avoid over-fitting and maintaining a sufficient number of lags. The delay between ECoG activity and the arm movement was found by varying *t*_0_ iteratively starting from a value of 100 ms—a figure reported as the latency between cortical motoneuron activity and emergence of EMG [42–45]—in steps of 5 ms until the highest accuracy was achieved. At each iteration, we measured the accuracy of the multilinear regression model using a leave-one-out method, calculating the correlation between the predicted and actual arm movements.

**Fig 7:**
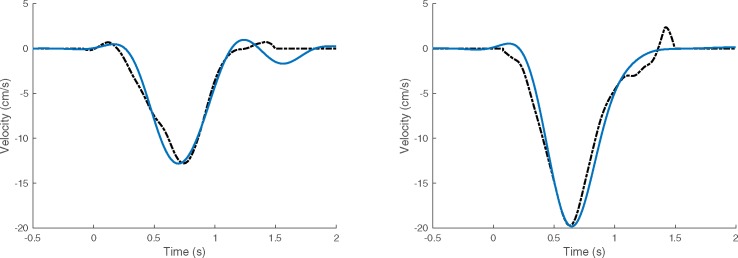
Arm velocity predicted by the multiple linear regression model. Prediction (solid line) and actual velocity (dotted line) over two different trials of reaching right (x-axis component) for Participant 3.

Predicted velocity was measured for each target separately. The average correlation between the predicted and actual velocities ranged between 78–95%. Table 3 details the accuracy for each participant. Typical reconstructed velocity profiles are shown in Fig 7 for Participant 3, where we see good agreement in the arm trajectories. Inspection of the coefficients suggests that the arm velocity is well predicted from the slow and gamma oscillations, but not from the alpha-beta activity. Most of the error appears after the arm has reached the target, when the participant is holding his/her hand in the air at the end of the trial. During this period, EMG activity was observed in all of the electrodes on the arm. The residual neural activity after movement cessation is likely to be attributed to maintaining the arm/hand posture against gravity.

We also looked at further generalizing our findings. First, we explored the possibility of using a model created using reaching to the right movements to reconstruct reaching to the left. The following steps were taken. First, the MLR model was trained using all of the trials corresponding to reaching to the right. Next, we used this model to process reaching to the left. The average correlation between the recorded value of the arm speed and the predicted values was 81%, which is very close to the accuracy obtained when the MLR model was developed and tested using reaching to left trials only (83%). In other words, the MLR model is not necessarily specific to the direction of reach.

Encouraged by this, we also generated results in the case where all of the trials are pooled together irrespective of reach direction. This is expected to yield the poorest results due to the fact that any systematic variations between the different reach directions will be averaged out. Again utilizing leave-one-out analysis (i.e. training on the entire data set irrespective of direction before testing a single trial), we found correlations of 81%, 74%, and 70% for participants 1, 2, and 3. Thus, for higher accuracy we would recommend, instead of utilizing a single model for all directions, to do direction discrimination and keeping a separate model for direction.

## Discussion

### Significance and comparison with previous studies

In this paper, we presented a method for detecting onset of movement with near perfect accuracy using ECoG recordings obtained from the primary motor cortex. This performance is higher than accuracies reported in earlier studies [7,8,27] despite using very few epidural ECoG contacts located over the sensorimotor cortex. The high prediction accuracies were achieved after defining frequency bands specific to each individual by selecting regions which showed the highest level of change during movement. Inspection of the model suggests that gamma activity is the best indicator of movement onset as this frequency band shows the largest difference between movement and rest activity. In addition to movement onset, we showed that it is possible to reconstruct upper limb velocity during reaching tasks. Our approach used a multilinear regression model to reconstruct the movement speed from ECoG activity. The average correlation between the actual and predicted arm velocity exceeded 80%. Although we explored reaching to only a limited number of targets, we believe that these results can be generalized to other reaching directions based on directional tuning of cortical activity [13,17,19,26,27,33,48,49]. Inspection of the model coefficients showed that upper limb velocity was best reconstructed from both the upper gamma band activity (60–100 Hz) and slow oscillations (<2 Hz). Prediction was much poorer using low gamma or the alpha-beta bands (12–60 Hz). This conclusion is consistent with some earlier studies [13,14,19,20,27].

In our study, movement classifiers were determined on a per subject basis by searching out bands where the most significant movement-related ECoG activity was observed. This is contrasted with the use of preset frequency bands determined without the consideration of cross-subject variability [13,41]. Measuring ERS’s/ERD’s over wide predefined bands reduces the discriminability of movement-related changes (Figs 3 and 6). To deal with this issue, some studies have divided the spectrum into a sequence of bands of fixed width [14,50]. For example, Pisthol et al. [14] studied the relationship between ECoG amplitude and arm kinematics by evaluating activity with bandwidth of 5 to 95 Hz in steps of 10 Hz. We introduced a more modest approach in this paper by using a 4 Hz band centered at the frequencies where the highest movement-related activities were found. This approach reduced the number of input parameters of the model and simplified the fitting process. Individualized selection of frequency bands increased the accuracy of our models both in terms of movement detection and reconstruction of arm kinematics. While our results were generated from electrodes located over the motor as well as the sensory cortex, the correlation between predicted and measured velocities did not changed appreciably when only the electrodes located over the motor cortex were used (p>0.05, Ranksum test).

Despite recording ECoG activity with only eight contacts, the prediction accuracy achieved exceeded those obtained by earlier studies with wider electrode coverage of cortical areas [13,14,16,17]. Schalk et al. [13] investigated circular tracking movements with a relatively restricted range. Their study yielded an average correlation of approximately 50% for arm trajectory. Pistohl et al. [14] extended the investigations to less restricted, target-directed, full two dimensional joystick movements. They showed that arm trajectory can be predicted from the low frequency components of ECoG signals with 43% correlation on average. Pistohl et al. also concluded that using the energy of other frequency bands (e.g. 40–80 Hz) does not significantly improve the prediction of the hand position [14]. Moreover, the same group found an average delay of approximately 90 ms in brain activity from the onset of movement. This value is similar to the delay we found in our analysis between the initiation of the cortical activity and movement onset.

The reconstruction of hand kinematics in our study had a similar performance to kinematics obtained from intra-cortical microelectrode recordings [11,12]. Wu et al. used a Kalman filter to decode two-dimensional trajectories from 42 single neuronal units [11]. They obtained an average correlation coefficient of 88% for hand position, similar to the prediction accuracy we obtained from only 8 ECoG contacts (85%). However, recording from single neuronal units continues to be a difficult task in humans due to the potential risks associated with the implantation procedure and system maintenance.

### System feasibility

The electrodes used in this study are commercially available and routinely implanted in neurological patients. They have been clinically validated for their stability and reliability. The current configuration has a number of advantages when compared to other intracranial brain recording techniques: 1) the ECoG electrodes do not penetrate the cortical surface thereby reducing the potential risk for tissue damage; 2) ECoG reflects population activities offering a better prospect for long-term recording stability when compared to single unit recordings; and 3) ECoG requires neither a high sampling rate nor spike detection/sorting capabilities; hence, the overall computational requirements are considerably reduced. We reconstructed the arm movement using 8 ECoG contacts with only two contacts placed on the motor cortex. Using a small number of contacts simplifies the system and results in less computational cost as well as shorter setup time. Moreover, the model used in this study is a simple linear model that requires little computational resources to estimate arm velocity. Such computations can be carried out on low-power processors. We believe that this setup is appropriate for real-time and clinical application for the development of an ECoG-based BMI system.

### Encoding of jerk in ECoG activity

An organizational principle for motor control has been proposed following the observation that arm movements tend to follow a path or trajectory that minimizes jerk [51,52]. Given the start and end points of the movement as well as its duration, a trajectory of minimal jerk can be found by minimizing

$$\int_{t_{s}}^{t_{e}}{\left|\vec{J}(t)\right|}^{2}dt$$

where $\vec{J}(t)$ is the first derivative of acceleration, and *t*_*s*_ and *t*_*e*_ are movement start and end times respectively. To our knowledge, no study has examined jerk minimization in the context of brain recordings. If the motor system does in fact obey such a principle, one would expect jerk to be reflected in the neural response underlying a reach task. One possibility is that neural activity encodes jerk and not velocity as was assumed in the multilinear model presented earlier.

What we found is that the results of our study do not reject the possibility that the nervous system encodes jerk. We draw this conclusion from the fact that arm velocity was found to be well predicted by the ECoG response. Since the velocity function is a bell-shape curve, two derivatives of this trajectory (for jerk) will yield another bell-like curve. Thus velocity, and jerk, can both be well-predicted from a multilinear ECoG model. To pursue this matter further—to better tease apart whether jerk or velocity is encoded—one would require a more complex motor task for which the velocity and jerk trajectories do not overlap.

### Study limitations

There were several limitations to the work presented in this study. First, the number of participants was small. This was due to the fact that only a limited number of individuals undergo implantation of ECoG electrodes specifically targeting the motor cortex each year. Direct stimulation of the motor cortex is not a routine intervention for treatment of pain thereby limiting the number of potential participants for this study. Second, there were a number of unique challenges with subject recruitment including: (1) that the measurements could only be performed in patients whose electrode contacts were externalized (with not many patients belonging in this group), (2) the contacts were externalized for a short period of time following surgery, and (3) some patients may not have felt well enough to participate in the research study after surgery. Third, the placement and choice of number of ECoG contacts in our study were dictated by clinical requirements unrelated to the purpose of this study. Indeed, the electrode placement may not have been optimal as coverage over the premotor cortex was missing. Activity from the premotor cortex has been found to be indicative of movement onset and direction [27].

## Conclusions

We have detailed a system which allows for high accuracy detection of movement onset and kinematic reconstruction of upper arm movement using a clinically realizable electrocorticographic-based system with only eight contacts placed over the sensory-motor cortex. Our results indicate that changes in gamma activity are the most reliable indicator of movement onset. Both low frequency (< 2 Hz) and gamma band cortical activities were used to develop a linear model which, in turn, allowed the reconstruction of arm velocity during reach tasks with accuracy exceeding 85%. Our approach differs from that of earlier studies due to the adaptive selection of frequency bands for use with movement classification and prediction on an individual subject basis.
